# Economic evaluations of psychological treatments for common mental disorders in low- and middle-income countries: protocol for a systematic review

**DOI:** 10.1080/16549716.2021.1972561

**Published:** 2021-09-12

**Authors:** Vimbayi Mutyambizi-Mafunda, Bronwyn Myers, Katherine Sorsdahl, Esther Chanakira, Crick Lund, Susan Cleary

**Affiliations:** a Health Economics Unit, School of Public Health and Family Medicine, University of Cape Town, Cape Town, South Africa; b Curtin enAble Institute, Faculty of Health Sciences, Curtin University, Perth, Australia; c Alcohol, Tobacco and Other Drug Research Unit, South African Medical Research Council, Cape Town, South Africa; d Division of Addiction Psychiatry, Psychiatry and Mental Health, University of Cape Town, Cape Town, South Africa; e Alan J Flisher Centre for Public Mental Health, Department of Psychiatry and Mental Health, University of Cape Town, Cape Town, South Africa; f School of Health and Related Research, University of Sheffield, Sheffield, UK; g Centre for Global Mental Health, Health Service and Population Research Department, Institute of Psychiatry, Psychology and Neuroscience, King’s College, London, UK

**Keywords:** Common mental disorders, psychological treatment, economic evaluation, low-middle income countries

## Abstract

**Background:**

Common mental disorders (CMDs) are highly prevalent conditions that constitute a major public health and economic burden on society in low- and middle-income countries (LMICs). Despite the increased demand for economic evidence to support resource allocation for scaled-up implementation of mental health services in these contexts, economic evaluations of psychological treatments for CMDs remain scarce.

**Objective:**

The proposed systematic review aims to synthesize findings on methods and outcomes of economic evaluations of psychological treatments for CMDs in LMICs and appraise quality.

**Methods:**

We will identify, select, and extract data from published economic evaluations of psychological interventions for CMDs conducted in LMICs. We will search bibliographic databases (PubMed, EMBASE, CINAHL, Web of Science, EconLit, PsycINFO, Africa-Wide Information, Cochrane library, Centre for Reviews and Dissemination (CRD), Cost Effectiveness Analysis (CEA) Registry), and the African Journals Online (AJOL) and Google Scholar platforms. Only full economic evaluations (Cost-Effectiveness Analysis (CEA), Cost-Utility Analysis (CUA), Cost-Consequence Analysis (CCA), or Cost-Benefit Analysis (CBA)) of psychological treatments for CMDs (defined as depressive, anxiety, and substance use disorders) conducted in LMICs will be included. There will be no restrictions based on date of publication, perspective, follow-up duration or sample size. Data extraction will be guided by the Consolidated Health Economic Evaluation Reporting Standards (CHEERS) checklist.

**Results:**

The results presented will be examined using a narrative synthesis approach. The quality of included studies will be assessed using the Drummond & Jefferson checklist.

**Conclusion:**

The fledgling evidence base in this area provides an opportunity to promote improved economic evaluation methods in line with repeated calls for economic evidence alongside effectiveness evidence in these settings. A rigorously developed economic evaluation evidence base will support resource allocation decisions for scaled up implementation of psychological interventions in LMIC settings.

**Systematic review registration:**

PROSPERO CRD42020185277.

## Background

Common mental disorders (CMDs) are highly prevalent conditions globally [1–3]. As a subset of Mental Neurological and Substance Use (MNS) disorders [4–7], CMDs are a significant contributor to disability worldwide [8,9]. Recent global health estimates show that these disorders accounted for 63% to 69% [10] of disability attributable to mental and substance use disorders across low- and middle-income countries (LMICs). CMDs are therefore a significant contributor to the global *economic* burden attributable to non-communicable diseases. This burden is projected to reach USD 6 trillion by 2030 [11,12]. Psychological treatments are promoted by the World Health Organisation (WHO) as first line treatments for CMDs [13], with evidence suggesting that these treatments have enduring effects superior to continued pharmacotherapy in high-income countries (HICs) [14]. Although the large global economic burden of mental illness is disproportionately carried by LMIC populations [11], the economic evaluation evidence needed to inform resource allocation [6,15,16] remains limited in these settings.

## Problem statement

Despite an established evidence base on the effectiveness of psychological treatments for CMDs [17] and numerous efforts to scale up services in LMICs [18,19], access to these treatments remains limited due to insufficient resources allocated to mental health care. Robust data on the costs, outcomes, and cost-effectiveness of different types of treatment (e.g. Cognitive Behavioural Therapy (CBT), Behavioural Activation (BA), Interpersonal Therapy (IPT) and Problem-Solving Therapy (PST)) is largely lacking. This limitation extends to different service delivery models including brief treatments delivered through task-sharing with trained non-specialist health workers (NSHW) [13,20], collaborative and stepped care approaches incorporating limited use of highly skilled providers especially in patients with co-morbidities [21–23], and delivery of treatments using mobile and online m(Health) applications [24–26]. As an example, in a broad global scenario analysis of economics and mental health, Knapp and Wong (2020) [12] highlighted the lack of cost-effectiveness data on BA treatments. The effectiveness of BA in comparison to widely used CBT is less dependent on the skills of the therapist therefore making it a potential option for treatment scale up in LMICs where specialist mental health care workers are scarce. These authors also noted the limited cost-effectiveness data on antenatal psychosocial interventions delivered through task-sharing using NSHW despite established effectiveness [12]. In addition to the need for more economic data, it is necessary to examine the methodological quality of existing data. It is unclear whether existing economic evaluations are methodologically rigorous, pointing to the need for an inspection of the methodological quality of the available literature.

While there are many reviews highlighting the methodological challenges associated with economic evaluations of psychological treatments for a range of CMDs, these are based almost exclusively on studies conducted in HICs. Given the scarcity of financial resources and specialist health care providers in LMIC settings [27,28] and other contextual factors [29], findings from HICs may not be generalisable to LMICs and there may be additional methodological challenges associated with economic evaluations in these settings. Levin and Chisholm (2016) [30] conducted a wide ranging review of the cost effectiveness and affordability of prevention and treatment interventions across the spectrum of MNS disorders in LMICs. However, to the best of our knowledge, there have been no reviews of economic evaluations of psychological treatments for CMDs (operationally defined as depressive, anxiety, and substance use disorders) with a methodological focus in LMICs. Given these limitations on the existing evidence, we propose a systematic review examining the methods and quality of these economic evaluation studies.

## Aims and objective(s)

The overall aim will be to systematically review economic evaluations of psychological treatments for CMDs in LMICs. The review will aim to answer the following research questions: what are the methods used in these economic evaluations; what is the quality of these economic evaluations; are valid conclusions drawn from these economic evaluations; do they support or limit decision making; and how can economic evaluation methods be improved to support resource allocation decisions for psychological treatments for CMDs? To answer these questions, the primary objectives of the review are to:

- summarize methods and outcomes of economic evaluations of psychological treatments for common mental disorders in LMICs, and
- appraise the quality of published studies.

As a secondary objective, the review will:

- critically examine the usefulness of the evidence base for informing resource allocation for psychological treatments for CMDs in LMICs.

It is hoped that addressing these evidence gaps will strengthen the translation of economic evaluation research into policy and support scale up of appropriate psychological treatment services for CMDs in LMIC settings.

## Economic evaluation evidence and methodological challenges

In the resource constrained settings of LMIC health systems, decisions to invest in treatments for CMDs are also informed in part by their comparative *cost*-effectiveness in relation to other interventions. This context of competing demands for health care resources ideally results in health care dollars being directed to those interventions that reflect efficiency in maximizing health, within the prevailing health care budget constraint [31]. Robust economic evaluations are needed to inform decision-making in such a competitive environment. This evidence needs to be developed using methods that promote transparency, comparability, and generalizability [32]. Such evidence is essential to support the delivery and implementation of psychological treatments at scale [19]. The application of robust methods for conducting and reporting economic evaluations may increase the extent to which psychological treatments for CMDs are considered for inclusion in benefits packages. The increase in intervention trials for psychological treatments of CMDs in LMICs [33] therefore needs to be accompanied by more rigorous economic evaluations [12]. The kinds of evidence required by decision makers can stem from various types of economic evaluations. The most common are detailed in Table 1, adapted from Drummond et al. (2015) [32].

**Table 1.** Main types of full economic evaluations*

Type of evaluation	Measurement/ valuation of costs in both alternatives	Identification of consequences	Measurement/valuation of consequences	Explanatory Notes	Policy Applications
Cost effectiveness analysis (CEA)	Monetary units	Single effect of interest, common to both alternatives, but achieved to different degrees.	Natural units (E.g. depression free days (DFDs), change in depression symptom severity, points of blood pressure reduction, etc.)	Provides useful evidence for comparing costs and outcomes for similar treatments using the same clinical outcomes. For example comparing CBT vs B.A as psychotherapeutic treatment for depression, outcome measure depression symptom severity measured using the Patient Health Questionnaire (PHQ-9) in Sun et al. (2021) [34].	Informs resource allocation within mental health budget for similar treatments that are evaluated using the same measure of outcome. Decision making tool: ICER [35]
Cost-utility analysis (CUA)	Monetary units	Single or multiple effects, not necessarily common to both alternatives.	Healthy years (typically measured as quality adjusted life-years (QALYs))	A form of CEA where outcomes are presented as multi-attribute outcomes such as disability adjusted life years (DALYs) and quality adjusted life years (QALYs). QALYs for example consider contributions of interventions to quality of life *and* length of life thus assisting with global budgetary decisions around how to best allocate health resources to maximize population health, All types of interventions for different conditions can be comparable due to the generic outcome measure. E.g. Sun et al. (2021) [34]	Informs resource allocation across health programs within national health budgets. Decision making tool: ICER and cost-effectiveness threshold [36]
Cost-benefit analysis (CBA)	Monetary units	Single or multiple effects not necessarily common to both alternatives	Monetary units	Ideologically appealing due to the potential to assign a monetary value to the many benefits of mental health treatments experienced across different sectors of society e.g. increased productivity in the workplace, reduced burden on social welfare, reductions in recidivism. However CBAs are difficult to conduct and generalize due to the complexity associated with monetizing benefits and determining the scope of these benefits. e.g. Iijima et al. (2013) [37] & Layard et al. (2007) [38]	Informs resource allocation across multi-sectoral budgets. Decision making tool: net benefits, cost benefit ratio (CBR), benefit cost ratio, return on investment (ROI) [39,40]

*Full economic evaluations compare costs and outcomes across at least 2 competing interventions

Adapted from Drummond et al. (2015) [32]

Various methodological concerns have been raised around the quality and usefulness of data generated mostly from economic evaluations of psychological treatments in HIC. These include questions about the breadth and quality of cost data used in economic evaluations [41–43]; the perspective applied [44–46]; appropriateness of time horizons [47–49]; and the application of multi-attribute outcome measures [46,47,49–51]. These issues are defined and briefly discussed in Table 2:

**Table 2.** Key economic evaluation definitions [52–56]

Term	Definition
Perspective	The viewpoint or approach taken in costing. A health system or provider perspective includes costs incurred by the provider (which could include a health care or other provider). A patient perspective considers patient, household and societal impacts not born by the provider, such as opportunity costs to patients, lost income by caregivers and productivity losses. A societal perspective includes both provider and patient perspectives.
Time horizon	The time frame over which costs and outcomes associated with an intervention are assessed.
Incremental Cost-Effectiveness Ratio (ICER)	ICER is the ratio of incremental costs to incremental outcomes, with incremental costs as the numerator and incremental outcomes as the denominator. In decision making an intervention with a lower ICER is generally preferred to one with a higher ICER as it is an indication of a lower additional cost per unit of gain.

### Cost data

The quality of cost data remains a well-recognized challenge for economic evaluations of mental health services. Previous reviews have highlighted problems associated with: unclear descriptions of cost categories, ambiguous definitions of cost components, and poor differentiation between patient and provider costs [43,50]. The limited scope of costs assessed is also problematic, with studies reporting the absence of intervention development costs, inpatient costs, and indirect costs [43,44,46,48,49,51,57]. While there is some debate about whether productivity changes should be defined as indirect costs or benefits [32], for the purposes of this review, we will denote them as indirect costs, following the Consolidated Health Economic Evaluation Reporting Standards (CHEERS) recommendations [58]. One review noted the importance of presenteeism as a productivity loss in depression [50]. The inclusion of *both* presenteeism and absenteeism in measuring lost productivity costs was a methodological enhancement suggested for CEAs of psychological treatments for depression [50]. Another review of depressive disorders showed that productivity changes contributed up to 60% of the total cost per treatment arm [59] while 70% of the studies reviewed did not report these costs.

### Perspective

Some reviews noted that a few studies did not clearly report their perspective [44,45]. Other reviews recommended the inclusion of a societal perspective to account for indirect costs such as productivity losses [46,49]. Many reviews identified studies reporting a societal perspective without including productivity losses as indirect costs [44,47,50]. These observations suggest that although the societal perspective can capture the wider costs and outcomes of these interventions [45,48,49], there are methodological challenges.

### Time horizon

When short-term time horizons are applied in evaluations it is difficult to understand the longer-term costs and outcomes of an intervention. This methodological limitation is a concern given the recurrent and chronic nature of some CMDs [45,46,48,49,51]. One review noted that studies with longer time horizons reported lower ICERs, whilst another showed that cost effectiveness results were reversed over longer time horizons [12,45].

### Multi-attribute outcomes

Another concern is the limited use of multi-attribute outcomes, which together with clinical outcomes provide useful information for planning and decision making using ICERs [12,32]. Economic evaluations of psychological treatments commonly apply clinical outcomes to measure health gain. Several reviews advocated for the use of multi-attribute outcomes in addition to clinical outcomes to improve comparability of cost effectiveness results and support decision making. The need to include QALYs to enable cost per QALY comparisons was noted in several reviews [46,47,49,51,60,61]. One review discussed the need to apply relevant and up to date cost effectiveness thresholds when assessing value for money [47].

Other methodological concerns highlighted in previous reviews include the limited use of sensitivity analysis [50,51], studies being inadequately powered to accurately measure costs [57,60] and ambiguity around management of missing data [48]. The highlighted methodological concerns were drawn from reviews of HIC evidence. Reviews considering economic evaluation evidence for psychological treatments in LMICs are limited, but they all emphasise the paucity of economic evidence [12,17,20,62–64]. As such, it is not clear whether the quality and scope of cost data, perspectives considered, time horizons, health outcomes and methods applied for estimating cost effectiveness provide information sufficient to support decision making in these settings.

In addition to the challenges highlighted in developing sound cost and outcome data, methodological inconsistencies in reporting and interpreting results may influence investment decisions and may contribute to the underinvestment in mental health treatments identified in various reviews [9,12]. Investments in psychological treatments for CMDs have benefits for individuals and society at large [65]. Although these investments are typically made from health sector budgets, the savings are also enjoyed by many other sectors that are impacted by untreated CMDs, such as: social welfare (increased productivity and less dependence on welfare), education, legal (less recidivism), safety and security (fewer accidents, less interpersonal violence). The consequence of this ‘diagonal accounting’ (Knapp & Wong, 2020) [12] is mental health ‘investment inertia’ on the part of the health sector. Health sector investments are informed by cost per QALY/DALY comparisons, favouring interventions which result in sharp and rapid reductions in mortality and morbidity at low cost i.e. interventions that at the population level are efficient in translating health dollars into health outcomes. The overall result is underinvestment in mental health especially psychological interventions, which are resource intensive, reflect outcomes that often manifest slowly over time and with benefits that spill over into other sectors. An examination of CBA evidence is therefore warranted as these types of economic evaluation consider the impacts of treatments across various sectors. This type of evidence has the potential to strengthen political arguments in favour of shifting national budgets to mental health services. Methodological rigor and consistency in economic evaluation may provide transparent and generalizable data to policy makers and influence resource allocation decisions.

In summary, findings from reviews suggest that the results of economic evaluations may be more useful to resource allocation decisions if broader and more detailed information on costs, appropriate time horizons and multi-attribute outcomes are included, and if the interpretation of results is harmonized. These methodological improvements would especially enhance evidence from trial based economic evaluations.

## Methods

This protocol is reported in line with Preferred Reporting Items for Systematic Reviews and Meta-Analysis (PRISMA-P) guidelines (Additional file 2) [66]. This review will be informed by the guidelines for reviews of economic evaluations published by the Cochrane Collaboration for Reviews and Centre for Reviews and Dissemination (CRD) [67].

### Eligibility criteria

Inclusion and exclusion criteria are listed in Table 3. Full economic evaluations (CEA, CUA, CBA) comparing the costs and outcomes of at least two alternatives will be included. In addition, we will include Cost-Consequence Analysis (CCA), which is very similar to CEA except that a range of health and non-health costs and outcomes are reported in a disaggregated manner [68]. We will include studies that collected primary data from patients including trial, non-trial, and quasi-experimental (non-randomised) studies. While it is common for economic evaluations to include secondary data (e.g. on unit costs or disability weights), modelled studies based entirely on secondary data will be excluded from this review as the breadth of these studies generally prevents an in-depth understanding of key methods. As the objective is to review the methodological quality of economic evaluations assessing results against standardised metrics for decision making such as incremental cost effectiveness ratios (ICERs), cost-analysis, cost-of illness, and budget- impact analyses will be excluded.

**Table 3.** Inclusion and exclusion criteria applied

Parameter	Include	Exclude
Economic Evaluation	Cost-Effectiveness Analysis Cost-Utility Analysis Cost-Consequence Analysis Cost-Benefit Analysis	Cost Analysis Cost-of-illness Budget-Impact Analysis
Design	Quantitative economic evaluation studies Cross sectional or longitudinal primary data collected from trial, non-trial, and quasi-experimental (non-randomised studies) Article reporting economic evaluation only or reporting economic evaluation results as part of an effectiveness trial manuscript Published in peer reviewed journals only	Qualitative studies/study protocols Modelled studies based entirely on secondary data Reviews/ short notes/editorial notes/opinion pieces/commentaries Conference abstracts/ book chapters/dissertations/grey literature
Participants/population	Patients with CMDs* only or with CMDs plus co-morbidities Studies where CMDs are identified using screening instruments and formal diagnoses	Studies that do not report results for CMD conditions separately from other conditions
Intervention(s) and exposure(s)	Psychological treatments for CMDs Psychological treatment as the primary therapy used in conjunction with pharmacotherapy as adjunctive treatment	Treatments that focus primarily or exclusively on the provision of pharmacotherapy only
Comparator(s) control	Full economic evaluation studies where comparators are stated. Examples of comparators may include: No intervention/Standard of care/Treatment as usual/Another intervention.	Studies reporting only costs or only outcomes or costs and outcomes of only one option without a comparator
Outcome	Studies reporting economic evaluation outcomes including natural/clinical and/or multi-attribute outcomes such as QALYs and DALYs	Studies reporting screening or prevention as the outcome rather than treatment related outcomes
Region	Low and middle income countries as defined by the World Bank as at June 2019	High income countries
Dates	Published before 30 June 2021	Published after June 2021
Language	Results presented in the English language	Results presented partly or fully in languages other than English

* CMD operational definition: depressive disorders, anxiety disorders and substance use disorders

In addition, study protocols, qualitative studies, reviews, conference abstracts, short notes, opinion pieces, editorial notes, grey literature, book chapters, and dissertations will be excluded from this review.

### Condition or domain to be studied

CMDs are the conditions to be studied. For the purposes of this review, we will use an operational definition of CMD conditions, which comprises: depressive disorders, anxiety disorders and substance use disorders [3–5,7,17,69]. Depressive disorders include major depressive disorder and dysthymia [5]. Anxiety disorders encompass generalized anxiety disorder (GAD), panic disorder, phobias, social anxiety disorder, obsessive-compulsive disorder (OCD) and post-traumatic stress disorder (PTSD) [5]. Substance use disorders include alcohol use disorders (AUDs) (harmful alcohol use, alcohol abuse, and alcohol dependence) [70] and drug use disorders (DUDs).

### Participants/population

The population under consideration is patients with CMDs, this will include people identified using screening instruments and formal diagnoses. We will exclude studies where patients with CMD conditions are combined with other conditions and the CMDs are not reported separately in analyses. There will be no restrictions on co-morbidity status, gender, or age of patients treated. We will only include studies from countries that were categorised as LMICs by the World Bank as of June 2019 [71].

### Intervention(s) and exposure(s)

Any study that includes treatments for CMDs that are primarily psychological in nature will be reviewed. This includes interventions where these psychological treatments are the primary therapy used in conjunction with pharmacotherapy as an adjunctive treatment. These psychological treatments typically involve different combinations of a variety of cognitive and behavioral approaches so are likely to be diverse; the findings will be compared and contrasted where relevant. Treatments that focus primarily or exclusively on the provision of pharmacotherapy will be excluded.

### Comparator(s) control

Only full economic evaluations where a comparator is stated will be reviewed. There will be no restrictions on the types of comparator(s).

### Outcomes

#### Primary outcomes

Outcome measures used in economic evaluations include natural units or multi-attribute outcome measures or both. In general, CEA uses clinical outcomes while CUA uses multi-attribute outcomes such as disability-adjusted life years (DALYs) or quality-adjusted life years (QALYs). Although there are many outcomes for the psychosocial treatment of CMD conditions, there will be no restrictions on study outcomes reported. This is in line with the purpose of the review, which is to assess methodology including outcomes that are reported in the economic evaluations of CMD psychological treatments.

#### Secondary outcomes

All outcomes as mentioned above.

### Other criteria

Only studies published in peer-reviewed journals where results are presented in the English language will be included. To minimize selection bias, there will be no restrictions based on perspective, follow-up duration, sample size or setting. To the best of our knowledge, this is the first systematic review of economic evaluations of psychological treatments for CMDs in LMIC settings, therefore we will include studies published before 30 June 2021, with no limitation back in time.

### Search strategy

We will search the listed databases to source potential studies for inclusion in the review (Table 4). We will also search the African Journals Online (AJOL) and Google Scholar platforms. We will hand search reference lists of included articles for additional articles that meet the inclusion criteria.

**Table 4.** List of databases to be searched

Bibliographic Databases to be searched	
PubMed (including Medline)	✓
EbscoHost (APA-PsycINFO, EconLit, CINAHL (Cumulative Index to Nursing and Allied Health Literature), Africa-Wide Information)	✓
Scopus (including EMBASE)	✓
Web of Science	✓
Cochrane Library (Cochrane Database of Systematic Reviews (CDSR) and Cochrane Central Register of Controlled Trials (CENTRAL)	✓
Cost-Effectiveness Analysis (CEA) Registry	✓
Centre for Reviews and Dissemination (CRD) (NHS Economic Evaluation Database (NHS EED))	✓

### Potential search terms

The search terms that we use will include: depression, anxiety disorders, alcohol disorders, psychological intervention, psychotherapy, psychosocial treatment, outcome assessment, economic evaluation, cost-effectiveness, comparative studies and LMIC. The draft search strategy for PubMed (Medline) developed with the support of a subject expert librarian is included as Additional file 1. Equivalent terms will be used to search in other databases.

### Study selection procedure

In conducting this review, we will use a two-stage approach. We will select papers using the stipulated inclusion and exclusion criteria. Initially, two independent reviewers will screen the titles and abstracts guided by the inclusion criteria. Following this, any disagreements will be resolved by a third reviewer. Full texts of the papers selected will then be accessed. These will again be reviewed for inclusion by two reviewers and a final decision will be made as to whether it should be included in the final synthesis.

A third author will be responsible for resolving any disagreements between reviewers, and this author will make the final decision on the eligibility of selected papers. Inter-rater agreement will be assessed by calculating the Kappa coefficient. We will pilot both stages of the selection process and modify where appropriate. In line with recommendations, we will develop a PRISMA [72] flow diagram to illustrate our study selection processes. Reasons for excluding articles will be documented in both stages of the selection process. Screening of articles will be done using Microsoft Excel® and references will be managed in Endnote 7 (Thomas Reuters) [73].

### Data extraction

The development of the data extraction form will be guided by CHEERS [58] which details 24 standard items which should be included when reporting an economic evaluation. If necessary, modifications will be made to capture data related to methodological challenges associated with economic evaluations of psychological treatments. As an example, we will extract data related to the measurement of treatment impacts including treatment group selection and attrition. We will also include specific fields to extract costs related to other referral and support systems for task-shifting, and where these are not provided, the review will critically reflect on the gaps in these costing data. The data extraction form will be populated in Microsoft Excel ®.

One reviewer (the first author) will extract the data using the extraction form. The second reviewer will independently validate the data extraction process by double checking the extracted data for completeness and accuracy. Discrepancies between the reviewers will be resolved through discussion. Where discrepancies cannot be resolved, a third reviewer will make the final decision.

### Quality assessment of included studies

We will use the Drummond and Jefferson [74] checklist to guide quality appraisal of published economic evaluations. The Drummond and Jefferson (1996) [74] checklist is available in short form (13 assessment criteria) and expanded form (35 assessment criteria) [32,74]. We will use the expanded version as it has the most comprehensive list of assessment criteria. It includes fields on study characteristics, quality and risk of bias, and other fields deemed methodologically important for economic evaluation. It also includes fields on productivity which is especially relevant to mental health evaluations. This checklist has been widely used in previous systematic reviews of mental health economic evaluations [43,44,49]. The same process detailed above for data extraction will be followed for the quality assessment. The reviewer (first author) will conduct the quality assessment and the second reviewer will independently validate for completeness and accuracy. Discrepancies between reviewers will be resolved through discussion with other co-authors.

### Strategy for data synthesis

In line with the CRD [67] and the Cochrane Collaborative [75] recommendations for reviews of economic evaluations, we will not be conducting a meta-analysis of quantitative findings. Economic evaluation studies are heterogenous in nature reflecting differences in settings, perspectives, time horizons, and measurements of costs and outcomes, so standard meta-analytical methods are considered inappropriate for synthesis. In addition, there is a lack of consensus on gold standard approaches to pooling combined estimates of cost data across economic evaluations. We will report on type of evaluation, perspective, outcomes, resource use, and sensitivity analysis. This will be done with a focus on the methods applied in identifying, measuring, and valuing of resources used (provider opportunity costs) and the scope of patient costs. The scope and valuation of outcomes will also be examined, including details on the measurement of treatment impacts to assess how these influence evaluation results. In addition to the granular examination of methods used to develop the cost and outcome data feeding into the evaluations, we will also present a narrative synthesis on the metric used to measure the relationship between costs and outcomes, for example the incremental cost effectiveness ratio (ICER) in CEA or the cost-benefit ratio (CBR) in CBA. The narrative synthesis will deal with the heterogeneous nature of economic evaluations by examining the interpretation of comparative cost/outcome results across studies and comparing the factors that influence these. Conclusions will be critiqued against economic evaluation guidance [58] and recent developments in assessing value for money [76–78] to determine whether cost-effectiveness interpretations are methodologically sound and reliable for guiding investment policy.

### Analysis of subgroups or subsets

Subgroup analysis will be considered where deemed appropriate focusing on the implications of economic findings for resource allocation [75]. Results may be grouped by CMD condition e.g. depression/anxiety/AUD. Other possible groupings of results include service delivery or organizational models (e.g. task-sharing or collaborative/stepped care), modes of treatment delivery (e.g. face-to-face or mHealth), and type of economic evaluation (e.g. CEA/CUA/CCA/CBA). Further sub-group analyses may include analyses by different age, gender, socio-economic groups, and settings characterised by conflict and violence given the correlations between effectiveness of psychological treatments and these domains.

### Reporting

Reporting of the review and its findings will be done in accordance with PRISMA guidelines [72]. The discussion will focus on the strengths and limitations of the methods used for economic evaluation. Methodological and reporting limitations and inconsistencies that compromise the robustness, reliability, generalizability, and transferability of economic evaluation results will be highlighted. Similar to other systematic reviews of economic evaluations, we will support the narrative synthesis with graphics and groupings that contribute to drawing useful implications for policy [43,46,61]. As mentioned, the Cochrane Collaborative highlights the importance of focusing on the implications of results (for policy) rather than meta-analytical synthesis when reviewing economic evaluations [75]. Therefore, the implications of the review findings will be discussed within the context of resource allocation for CMDs in LMICs.

## Discussion

Previous reviews have examined economic evaluations of interventions for mental, neurological and substance use (MNS) conditions [9,12]. The most comprehensive focused on the cost-effectiveness and affordability of a wide range of treatment and prevention interventions for MNS in LMICs and was conducted as part of the Disease Control Priorities (DCP) program by Levin and Chisholm (2016) [30]. This review noted the paucity of economic evidence from MNS interventional trials in LMICs and highlighted this as an impediment to country-level resource allocation decisions [9]. A recent broad scenario analysis by Knapp and Wong (2020) [12] discussed the current global context of mental health economics, highlighting key gaps in economic evaluation. Although not a primary focus, this review also highlighted the dearth of cost effectiveness evidence for psychosocial treatments delivered by NSHW [12]. Cubillos et al (2020) [64] noted that investments in programs for integrating behavioural health services into primary care using task-sharing and collaborative/stepped care approaches yielded cost-effective estimates in LMIC settings. The latter review also noted that cost savings were reported when a societal perspective was used in economic evaluations, pointing to the importance of inclusion of broader costs in these economic evaluations. Two reviews that focused on NSHW delivery in LMICs summarized economic outcomes as a secondary aspect [17,20] and highlighted the need for more economic evaluation evidence to support investments in this cadre of health workers. It is evident from this handful of reviews that good quality economic evaluations of first line psychological treatments for CMDs is necessary to support funding decisions.

The other, mostly HIC systematic reviews on economic evaluations of psychological treatments for CMDs focused on specific disorders, specifically depressive disorders [42,47–51,57,60,79], anxiety disorders [45,46], both anxiety and depressive disorders [22,43,61] and alcohol use disorders [80]. Some of these condition-specific economic evaluation reviews focused on the type of psychological treatment (most commonly CBT) [48,49,60], and organization of care (for example, collaborative or stepped care [22,47,51]) and electronic delivery mediums [43,44,49]. Most reviews were of cost-effectiveness analyses, with one specifically focused on CUA [48]. In the absence of a broad literature on economic evaluations in LMIC settings, these HIC reviews highlighted the possible methodological challenges associated with evaluations of psychological treatments.

We will build on the Levin and Chisholm [30] and Knapp and Wong [12] reviews by focusing on psychological treatments for CMDs (as defined) in LMIC settings. By using a methodological lens to highlight limitations and inconsistencies in applying economic evaluation methods, we will fill a gap in the evidence base, which currently has a HIC focus. Applying a methodological lens in a systematic review in an area of research where many effectiveness but limited economic studies are available may guide the development of a rigorous economic evaluation evidence base. This has been shown to be useful in informing resource allocation for other public health priorities [81]. Psychological treatments, recommended by the World Health Organization (WHO) as first line treatments for CMDs [13] are needed in LMIC settings where there is a large treatment gap but investment in these services is limited. Given that there are a number of effectiveness trials for CMD treatments in the pipeline, which have nested economic evaluations as part of their protocols [82–85], it is hoped that the proposed review will highlight gaps and guide improvements in the methods used in the emerging economic evaluation evidence base in these settings.

## Supplementary Material

Supplemental Material

## Paper context

Previous reviews examining economic evaluation evidence for Mental Neurological and Substance Use conditions in low- and middle-income countries have considered multiple conditions and interventions. To the best of our knowledge, this will be the first systematic review synthesizing methods and outcomes of economic evaluations for psychological treatments of Common Mental Disorders in these contexts. We will identify limitations and make suggestions for improving economic evaluations to better inform resource allocation decisions for scaling up treatment.
