# Supplementary material for: Economic evaluations of psychological treatments for common mental disorders in low- and middle-income countries: protocol for a systematic review
Source: Glob Health Action. 2021 Sep 12;14(1):1972561. doi: 10.1080/16549716.2021.1972561 (PMC8439217; doi:10.1080/16549716.2021.1972561)
Supplement: Supplemental Material [file ZGHA_A_1972561_SM7103.zip › Supplementary/ADDITIONAL FILE 1_SearchMEDLINE_REVISION-FINAL_06072021.docx]

**Additional File 1: Sample Search Strategy From MEDLINE**

**MEDLINE (PubMed)**

1. Depression [MeSH Terms]
2. Depressive disorder [MeSH Terms]
3. Anxiety disorders [MeSH Terms]
4. Drinking Behavior [MeSH Terms]
5. Substance-Related Disorders [MeSH Terms]
6. Stress Disorders, Post-Traumatic [MeSH Terms]
7. Depression [Title/Abstract] OR depressive [Title/Abstract] OR major depressive disorder [Title/Abstract] OR dysthymia [Title/Abstract] OR anxiety disorder [Title/Abstract] OR generalized anxiety disorder [Title/Abstract] OR social anxiety disorder [Title/Abstract] OR panic disorder [Title/Abstract] OR phobias [Title/Abstract] OR obsessive-compulsive disorder [Title/Abstract] OR OCD[Title/Abstract] OR Alcohol consumption[Title/Abstract] OR alcohol disorders[Title/Abstract] OR alcohol drinking[Title/Abstract] OR alcohol use[Title/Abstract] OR alcohol abuse[Title/Abstract] OR alcoholism[Title/Abstract] OR post-traumatic stress disorder [Title/Abstract] OR posttraumatic stress disorder [Title/Abstract] OR trauma [Title/Abstract] OR PTSD [Title/Abstract] OR Drug abuse [Title/Abstract] OR Drug dependence [Title/Abstract] OR Drug use Disorder [Title/Abstract] OR Drug Addiction [Title/Abstract] OR Substance use [Title/Abstract] OR Substance Dependence [Title/Abstract] OR Substance abuse [Title/Abstract]
8. 1 OR 2 OR 3 OR 4 OR 5 OR 6 OR 7
9. "Outcome Assessment, Health Care"[Mesh]
10. "Outcome Assessment, Health Care/economics"[Mesh]
11. "Primary Health Care/economics"[Mesh]
12. "Delivery of Health Care"[Mesh]
13. Costs[MeSH Terms]
14. Cost Analysis[MeSH Terms]
15. Cost[Title/Abstract] OR costs[Title/Abstract] OR expenditure[Title/Abstract] OR Benefit-to-cost[Title/Abstract] OR Cost benefit[Title/Abstract] OR Cost-benefit[Title/Abstract] OR cost-consequence[Title/Abstract] OR cost-effective[Title/Abstract] OR cost-effectiveness[Title/Abstract] OR cost-utility[Title/Abstract] OR economic evaluation[Title/Abstract] OR economics[Title/Abstract]
16. 9 OR 10 OR 11 OR 12 OR 13 OR 14 OR 15
17. Psychotherapy[MeSH Terms])
18. Psychosocial Support Systems[MeSH Terms]
19. Psychiatric Rehabilitation[MeSH Terms]
20. Mental Health Services[MeSH Terms]
21. Psychotropic Drugs[MeSH Terms]
22. Behaviour therapy[Title/Abstract] OR behavior therapy[Title/Abstract] OR brief interventions[Title/Abstract] OR Cognitive therapy[Title/Abstract] OR Counseling[Title/Abstract] OR counselling[Title/Abstract] OR group support[Title/Abstract] OR mental health intervention[Title/Abstract] OR mental health services[Title/Abstract] OR motivational interviewing[Title/Abstract] OR problem-solving therapy[Title/Abstract] OR psychiatric intervention[Title/Abstract] OR psychiatric services[Title/Abstract] OR psychosocial intervention[Title/Abstract] OR psychosocial support[Title/Abstract] OR Psychotherapy[Title/Abstract] OR psychotherapeutic[Title/Abstract] OR psychotherapies[Title/Abstract] OR psychological intervention[Title/Abstract] OR relaxation therapy[Title/Abstract] OR self-help groups[Title/Abstract] OR social support[Title/Abstract] OR meditation[Title/Abstract] OR mindfulness[Title/Abstract]
23. 17 OR 18 OR 19 OR 20 OR 21 OR 22
24. Clinical Trial[Title/Abstract] OR Comparative study[Title/Abstract] OR comparative studies[Title/Abstract] OR Controlled[Title/Abstract] OR evaluation study[Title/Abstract] OR evaluation studies[Title/Abstract] OR follow-up study[Title/Abstract] OR follow-up studies[Title/Abstract] OR longitudinal study[Title/Abstract] OR longitudinal studies[Title/Abstract] OR non-randomised[Title/Abstract] OR non-randomized[Title/Abstract] OR program evaluation[Title/Abstract] OR programme evaluation[Title/Abstract] OR prospective study[Title/Abstract] OR prospective studies[Title/Abstract] OR randomised[Title/Abstract] OR randomized[Title/Abstract] OR quantitative study[Title/Abstract] OR quantitative studies[Title/Abstract] OR quasi experimental[Title/Abstract] OR trial[Title/Abstract] OR trials[Title/Abstract] OR cohort[Title/Abstract]
25. Deprived Countries[Text Word] OR Deprived Population[Text Word] OR Deprived Populations[Text Word] OR Developing Countries[Text Word] OR Developing Country[Text Word] OR Developing Economies[Text Word] OR Developing Economy[Text Word] OR Developing Nation[Text Word] OR Developing Nations[Text Word] OR Developing Population[Text Word] OR Developing Populations[Text Word] OR Developing World[Text Word] OR LAMI Countries[Text Word] OR LAMI Country[Text Word] OR Less Developed Countries[Text Word] OR Less Developed Country[Text Word] OR Less Developed Economies[Text Word] OR Less Developed Nation[Text Word] OR Less Developed Nations[Text Word] OR Less Developed World[Text Word] OR Lesser Developed Countries[Text Word] OR Lesser Developed Nations[Text Word] OR LMIC[Text Word] OR LMICS[Text Word] OR Low GDP[Text Word] OR Low GNP[Text Word] OR Low Gross Domestic[Text Word] OR Low Gross National[Text Word] OR Low Income[Text Word] OR Lower GDP[Text Word] OR lower gross domestic[Text Word] OR Lower Income[Text Word] OR Middle Income[Text Word] OR Poor Countries[Text Word] OR Poor Country[Text Word] OR Poor Economies[Text Word] OR Poor Economy[Text Word] OR Poor Nation[Text Word] OR Poor Nations[Text Word] OR Poor Population[Text Word] OR Poor Populations[Text Word] OR poor world[Text Word] OR Poorer Countries[Text Word] OR Poorer Economies[Text Word] OR Poorer Economy[Text Word] OR Poorer Nations[Text Word] OR Poorer Population[Text Word] OR Poorer Populations[Text Word] OR Third World[Text Word] OR Transitional Countries[Text Word] OR Transitional Country[Text Word] OR Transitional Economies[Text Word] OR Transitional Economy[Text Word] OR Under Developed Countries[Text Word] OR Under Developed Country[Text Word] OR under developed nations[Text Word] OR Under Developed World[Text Word] OR Under Served Population[Text Word] OR Under Served Populations[Text Word] OR Underdeveloped Countries[Text Word] OR Underdeveloped Country[Text Word] OR underdeveloped economies[Text Word] OR underdeveloped nations[Text Word] OR underdeveloped population[Text Word] OR Underdeveloped World[Text Word] OR Underserved Countries[Text Word] OR Underserved Nations[Text Word] OR Underserved Population[Text Word] OR Underserved Populations[Text Word])) OR (Afghanistan[Text Word] OR Albania[Text Word] OR Algeria[Text Word] OR American Samoa[Text Word] OR Angola[Text Word] OR Armenia[Text Word] OR Azerbaijan[Text Word] OR Bangladesh[Text Word] OR Belarus[Text Word] OR Byelarus[Text Word] OR Belorussia[Text Word] OR Belize[Text Word] OR Benin[Text Word] OR Bhutan[Text Word] OR Bolivia[Text Word] OR Bosnia[Text Word] OR Botswana[Text Word] OR Brazil[Text Word] OR Bulgaria[Text Word] OR Burma[Text Word] OR Burkina Faso[Text Word] OR Burundi[Text Word] OR Cabo Verde[Text Word] OR Cape Verde[Text Word] OR Cambodia[Text Word] OR Cameroon[Text Word] OR Central African Republic[Text Word] OR Chad[Text Word] OR China[Text Word] OR Colombia[Text Word] OR Comoros[Text Word] OR Comores[Text Word] OR Comoro[Text Word] OR Congo[Text Word] OR Costa Rica[Text Word] OR Cote d'Ivoire[Text Word] OR Cuba[Text Word] OR Djibouti[Text Word] OR Dominica[Text Word] OR Dominican Republic[Text Word] OR Ecuador[Text Word] OR Egypt[Text Word] OR El Salvador[Text Word] OR Equatorial Guinea[Text Word] OR Eritrea[Text Word] OR Ethiopia[Text Word] OR Fiji[Text Word] OR Gabon[Text Word] OR Gambia[Text Word] OR Gaza[Text Word] OR Georgia[Text Word] OR Georgia Republic[Text Word] OR Ghana[Text Word] OR Grenada[Text Word] OR Grenadines[Text Word] OR Guatemala[Text Word] OR Guinea[Text Word] OR Guinea- Bissau[Text Word] OR Guyana[Text Word] OR Haiti[Text Word] OR Herzegovina[Text Word] OR Hercegovina[Text Word] OR Honduras[Text Word] OR India[Text Word] OR Indonesia[Text Word] OR Iran[Text Word] OR Iraq[Text Word] OR Ivory Coast[Text Word] OR Jamaica[Text Word] OR Jordan[Text Word] OR Kazakhstan[Text Word] OR Kenya[Text Word] OR Kiribati[Text Word] OR Democratic People's Republic of Korea[Text Word] OR Kosovo[Text Word] OR Kyrgyz[Text Word] OR Kirghizia[Text Word] OR Kirghiz[Text Word] OR Kyrgyzstan[Text Word] OR Lao PDR[Text Word] OR Laos[Text Word] OR Lebanon[Text Word] OR Lesotho[Text Word] OR Liberia[Text Word] OR Libya[Text Word] OR Macedonia[Text Word] OR Madagascar[Text Word] OR Malawi[Text Word] OR Malay[Text Word] OR Malaya[Text Word] OR Malaysia[Text Word] OR Maldives[Text Word] OR Mali[Text Word] OR Marshall Islands[Text Word] OR Mauritania[Text Word] OR Mauritius[Text Word] OR Mexico[Text Word] OR Micronesia[Text Word] OR Moldova[Text Word] OR Mongolia[Text Word] OR Montenegro[Text Word] OR Morocco[Text Word] OR Mozambique[Text Word] OR Myanmar[Text Word] OR Namibia[Text Word] OR Nepal[Text Word] OR Nicaragua[Text Word] OR Niger[Text Word] OR Nigeria[Text Word] OR Pakistan[Text Word] OR Palau[Text Word] OR Papua New Guinea[Text Word] OR Paraguay[Text Word] OR Peru[Text Word] OR Philippines[Text Word] OR Principe[Text Word] OR Romania[Text Word] OR Ruanda[Text Word] OR Rwanda[Text Word] OR Samoa[Text Word] OR Sao Tome[Text Word] OR Senegal[Text Word] OR Serbia[Text Word] OR Sierra Leone[Text Word] OR Solomon Islands[Text Word] OR Somalia[Text Word] OR South Africa[Text Word] OR South Sudan[Text Word] OR Sri Lanka[Text Word] OR St Lucia[Text Word] OR St Vincent[Text Word] OR Sudan[Text Word] OR Surinam[Text Word] OR Suriname[Text Word] OR Swaziland[Text Word] OR Syria[Text Word] OR Syrian Arab Republic[Text Word] OR Tajikistan[Text Word] OR Tadzhikistan[Text Word] OR Tajikistan[Text Word] OR Tadzhik[Text Word] OR Tanzania[Text Word] OR Thailand[Text Word] OR Timor[Text Word] OR Togo[Text Word] OR Tonga[Text Word] OR Tunisia[Text Word] OR Turkey[Text Word] OR Turkmen[Text Word] OR Turkmenistan[Text Word] OR Tuvalu[Text Word] OR Uganda[Text Word] OR Ukraine[Text Word] OR Uzbek[Text Word] OR Uzbekistan[Text Word] OR Vanuatu[Text Word] OR Venezuela[Text Word] OR Vietnam[Text Word] OR West Bank[Text Word] OR Yemen[Text Word] OR Zambia[Text Word] OR Zimbabwe[Text Word]))
26. 8 AND 16 AND 23 AND 24 AND 25
